# Impact of different management measures on the colonization of broiler chickens with ESBL- and pAmpC- producing *Escherichia coli* in an experimental seeder-bird model

**DOI:** 10.1371/journal.pone.0245224

**Published:** 2021-01-07

**Authors:** Caroline Robé, Katrin Daehre, Roswitha Merle, Anika Friese, Sebastian Guenther, Uwe Roesler

**Affiliations:** 1 Institute for Animal Hygiene and Environmental Health, Freie Universität Berlin, Berlin, Germany; 2 Institute for Veterinary Epidemiology and Biostatistics, Freie Universität Berlin, Berlin, Germany; 3 Institute of Pharmacy, Pharmaceutical Biology, Universität Greifswald, Greifswald, Germany; University of Connecticut, UNITED STATES

## Abstract

The colonization of broilers with extended-spectrum β-lactamase- (ESBL-) and plasmid-mediated AmpC β-lactamase- (pAmpC-) producing Enterobacteriaceae has been extensively studied. However, only limited data on intervention strategies to reduce the colonization throughout the fattening period are available. To investigate practically relevant management measures for their potential to reduce colonization, a recently published seeder-bird colonization model was used. Groups of 90 broilers (breed Ross 308) were housed in pens under conventional conditions (stocking of 39 kg/m^2^, no enrichment, water and feed *ad libitum*). Tested measures were investigated in separate trials and included (I) an increased amount of litter in the pen, (II) the reduction of stocking density to 25 kg/m^2^, and (III) the use of an alternative broiler breed (Rowan x Ranger). One-fifth of ESBL- and pAmpC- negative broilers (n = 18) per group were orally co-inoculated with two *E*. *coli* strains on the third day of the trial (seeder). One CTX-M-15-positive *E*. *coli* strain (ST410) and one CMY-2 and mcr-1-positive *E*. *coli* strain (ST10) were simultaneously administered in a dosage of 10^2^ cfu. Colonization of all seeders and 28 non-inoculated broilers (sentinel) was assessed via cloacal swabs during the trials and a final necropsy at a target weight of two kilograms (= d 36 (control, I-II), d 47 (III)). None of the applied intervention measures reduced the colonization of the broilers with both the ESBL- and the pAmpC- producing *E*. *coli* strains. A strain-dependent reduction of colonization for the ESBL- producing *E*. *coli* strain of ST410 by 2 log units was apparent by the reduction of stocking density to 25 kg/m^2^. Consequently, the tested management measures had a negligible effect on the ESBL- and pAmpC- colonization of broilers. Therefore, intervention strategies should focus on the prevention of ESBL- and pAmpC- colonization, rather than an attempt to reduce an already existing colonization.

## Introduction

Antibiotic resistance is an increasing threat to global public health and concerns human- and veterinary medicine [1]. Special attention is required for extended-spectrum β-lactamase (ESBL-) and plasmid-mediated AmpC β-lactamase- (pAmpC-) producing Enterobacteriaceae as they can limit therapeutic options with critically important antimicrobials, such as cephalosporins of 3^rd^, 4^th^ and 5^th^ generation as well as monobactams [2].

Broiler chickens are considered as a reservoir for ESBL- and pAmpC- producing bacteria, it with frequently confirmed high prevalence [3, 4]. These antibiotic-resistant bacteria are detected at every level of the broiler production chain. Comprehensive data on ESBL- and pAmpC- detection are available for parent- and grandparent stocks [5], the hatchery level [6], the fattening period [7, 8], the slaughterhouse level [9], and the commercial product [10]. Various transmission routes are described for ESBL- and pAmpC- producing bacteria. Dame-Korevaar et al. [11] reviewed transmission routes in the broiler production chain and categorized them as 1) vertical transmission between generations, 2) transmission at hatcheries, 3) horizontal transmission on the farm, 4) horizontal transmission between farms, and via the environment of farms. A transmission can lead to colonization of young broiler chickens through the oral uptake of the resistant bacteria. The spread of ESBL- and pAmpC- producing *Escherichia coli (E*. *coli)* in the broiler production chain can partly be attributed to the very low colonization dosage. Recent findings have shown that 10^1^ to 10^2^ colony forming units (cfu) of orally administered ESBL- and pAmpC- producing *E*. *coli* can already lead to colonization of broiler chickens [12, 13]. Cleaning and disinfection procedures can lower the risk of transmission of cephalosporin-resistant *E*. *coli* from one flock to the subsequent flock in broiler fattening farms [14]. Nevertheless, a reduction of the microbial load through intense cleaning and disinfection processes cannot eliminate ESBL- and pAmpC- producing bacteria and the recirculation of resistant bacteria from earlier production rounds could play a role in contaminating the consecutive flock [5].

Different measures to influence the microbial composition of the broiler chickens’ guts and to affect the colonization of broiler chickens with ESBL- and pAmpC- producing Enterobacteriaceae are discussed in literature. One approach is the direct modulation of the broiler chickens’ microbiota to influence the early colonization with ESBL- and pAmpC- producing bacteria. For example, probiotics such as commercial Competitive Exclusion cultures and phytobiotics showed promising results to reduce the ESBL- and pAmpC- colonization of broiler chickens [15–18]. Another approach is to modify the conventional housing conditions during the fattening process. A modification of the conventional conditions by an environmental enrichment is applied frequently in broiler production to increase animal welfare. However, information on the practical application and the economics of the production systems is often lacking certainty [19]. The conventional conditions for broiler production in Germany most commonly include the broiler breed Ross 308, no environmental enrichment during the fattening process, a stocking density of 39 kilograms per square meter (kg/m^2^), and water and feed *ad libitum*. Due to the variety of study designs, no clear statement on the impact of practically relevant management measures on the colonization of broiler chickens with ESBL- and pAmpC- producing Enterobacteriaceae is possible. We aimed to investigate different management measures which are taken during the fattening process on their potential to reduce the spread of ESBL- and pAmpC- producing bacteria. We investigated the replacement of the broiler breed Ross 308 with the broiler breed Rowan x Ranger, increase of the common litter quantity of one kg/m^2^ to three kg/m^2^, and reduction of the common stocking density of 39 kg/m^2^ to 25 kg/m^2^. In our study, the investigated management measures had a negligible impact on the ESBL- and pAmpC- colonization of the broiler chickens.

## Materials and methods

### Ethics statement

This study was carried out following the National Animal Protection Guidelines. The protocol was approved by the German Animal Ethics Committee for the protection of animals of the Regional Office for Health and Social Affairs Berlin (“Landesamt für Gesundheit und Soziales”, LAGeSo, permission number G 0193/16). All applicable national and institutional guidelines of the Freie Universität Berlin for the care and use of animals were followed. Experimental treatments of animals were classified as to lead to no worse than minor discomfort in the animals due to low pain of very short duration and were approved by LAGeSo.

### Housing conditions

All trials were set up successively in the experimental facilities of the Center for Infection Medicine of the Department for Veterinary Medicine, Freie Universität Berlin, using controlled rooms with respective ventilation and HEPA filtration of the exhaust air. Before each trial, related room was disinfected using hydrogen peroxide fumigation, and the absence of ESBL- and pAmpC- producing bacteria was confirmed. Before entering the room, daily clothes and shoes were changed with trial specific clothing in an attached separate anteroom.

**Fig 1:**
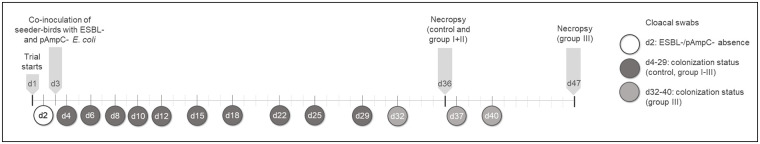
Study design of the experimental groups (control group, increased litter (I), reduced stocking density (II), and alternative breed (III)). d = day of trial.

In accordance with our recently established seeder-bird colonization model [12], a control group, having the broiler breed Ross 308 (mixed gender), was kept in a pen until the broilers reached a target weight of two kilograms (= 36 days (d) of trial). Ninety broiler chickens were housed under conventional conditions, with one-fifth of them (n = 18) being orally co-inoculated with 10^2^ cfu of one ESBL- and one pAmpC- producing *E*. *coli* on the third day of the trial (Fig 1). Said conventional conditions included a stocking density of 39 kg/m^2^ corresponding to 4.6 m^2^ for 90 broiler chickens, fresh litter once at the beginning of the trial (one kg/m^2^), no environmental enrichment, and conventional feed and water *ad libitum*. The light regime was set to 11 hours of light and 13 hours of dark with a dimming period of 30 minutes. The floor temperature was decreased from 28°C (d 1–6), over 26°C (d 7–13) and 24°C (d 14–20) to 22°C (d 21 to the end of the trial) with a relative humidity of 55%. The broiler chickens were fed a commercial starter feed (d 1–7), grower feed, and a finisher feed (five days before finalization of each trial) from a commercial broiler producer. Except for the finisher feed, coccidiostats were included in the feed (decoquinate and narasin/nicarbazin). Neither in the control nor the intervention groups antimicrobial agents were administered.

The intervention groups (I-III, Fig 1) were kept under the same conditions as the control group, altering only one management measure in each group. To evaluate their impact on the colonization of the broiler chickens with ESBL- and pAmpC- producing *E*. *coli*,

1. the litter quantity in kg/m^2^ was tripled from one kg/m^2^ to three kg/m^2^ over the course of the trial. We procured the litter which consisted of pelletized straw granules from a commercial broiler producer. The broiler breed (Ross 308, mixed gender), the number of broiler chickens (n = 90), the stocking density of 39 kg/m^2^, and the trial period of 36 days remained unchanged.
2. the common stocking density of 39 kg/m^2^ in Germany [20] was reduced to 25 kg/m^2^, as described in the Commission Regulation (EC) No. 543/2008 [21]. For this, the effective area of the experimental setup was enlarged from 4.6 m^2^ to 7.2 m^2^, whilst the broiler breed (Ross 308, mixed gender), the number of broiler chickens (n = 90), the litter quantity of one kg/m^2^ and the trial period of 36 days remained unchanged.
3. the broiler breed Rowan x Ranger (mixed gender) was included. In contrast to Ross 308, this breed is included in ‘slow-growing’ broiler concepts with an extended fattening period. The duration of the trial was set to 47 days so that the broilers reach the target weight of two kg. The number of broiler chickens per trial (n = 90), the stocking density of 39 kg/m^2^, and the litter quantity of one kg/m^2^ remained unchanged.

For each trial, we procured eggs from a commercial hatchery. The first disinfection using formaldehyde gas was already performed in the hatchery. After the transportation to the experimental facilities, the second liquid disinfection of the eggs was performed using WESSOCLEAN^®^ K 50 Gold Line containing 2.37% hydrogen peroxide and 0.015% peracetic acid (Wesso AG, Hersbruck, Germany). Following the second disinfection, the eggs for each experimental group were hatched in-house for 21 days. The eggs were incubated from day 1 to day 18 at 37.8°C and 60% relative humidity in a setter. From day 18 to day 21, the eggs were incubated at 37.2°C and 80% relative humidity for hatching.

Before the trial, the absence of ESBL- and pAmpC- producing bacteria was confirmed in the eggshells and in the one-day-old broiler chickens as well as in the experimental room and on the equipment as described in our seeder-bird colonization model [12].

### ESBL- and pAmpC- colonization strains

We used two avian *E*. *coli* strains to co-colonize our broiler chickens. One ESBL- producing *E*. *coli* strain (CTX-M-15; multilocus sequence type (ST) 410; phylogenetic group B1; internal number 10716) published as R56 by Falgenhauer et al. [22] and one pAmpC- producing *E*. *coli* strain (CMY-2, mcr-1; ST10; phylogenetic group A; internal number 10717). Both strains were isolated in a previous research project [23] and were recently used to establish our seeder-bird colonization model [12]. In this colonization model, both commensal *E*. *coli* strains colonized the digestive tracts of broilers with high bacterial counts and without causing any clinical signs.

The bacterial suspension for the inoculation of the broiler chickens with the ESBL- and pAmpC- *E*. *coli* strains was prepared, according to Robé et al. [12].

### Oral ESBL- and pAmpC- inoculation of the broilers

On the third day of trial, one-fifth of the broilers (n = 18) were orally co-inoculated with 10^2^ cfu *E*. *coli* of both bacterial strains in equal parts (Fig 1). These 18 seeder-birds were randomly selected prior to the trial and for the inoculation, 200 μl of the bacterial suspension were administered into the crop via a crop needle. After inoculation, the broiler chickens were immediately put back into the pen to the 72 non-inoculated broiler chickens (sentinel-birds). Regardless of the subsequent colonization status of the seeder-birds after inoculation, all broilers were kept together in one experimental setup to mimic real farming conditions.

### Samplings and analyses

The sample processing was identical to the one which was described in our seeder-bird colonization model [12]. Chromogenic orientation agar (CHROMagar Orientation, Mast Diagnostica, Reinfeld, Germany) was used for a reliable identification of the *E*. *coli* colonies. To confirm the ESBL-/ pAmpC- absence in the experimental room and the newly hatched broiler chickens, the agar was supplemented with two μg/ml cefotaxime (AppliChem, Darmstadt, Germany). In order to process all other samples, a set of four chromogenic agar plates which has proven suitable for our study was used. The total count of *E*. *coli* colonies was determined using an agar plate without selective media (positive control). For the detection of the ESBL- *E*. *coli* 10716, one plate was supplemented with two μg/ml cefotaxime and four μg/ml enrofloxacin (Sigma- Aldrich, Steinheim, Germany). For the detection of the pAmpC- *E*. *coli* 10717, one agar plate was supplemented with two μg/ml cefotaxime and seven μg/ml colistin (Carl Roth, Karlsruhe, Germany). The fourth agar plate contained all three antibiotics in the given concentrations (negative control). All samples were incubated for 24 h at 37°C. Every untypical *E*. *coli* colony morphology was further analyzed using MALDI- TOF (MALDI Microflex LT^®^ and Biotyper database^®^; Bruker Daltonics, Bremen, Germany).

#### Colonization status during the trial

The colonization of all 18 seeder-birds and 28 selected sentinel-birds with both *E*. *coli* strains was monitored over the entire period of each trial via cloacal swabs. The investigated birds were randomly selected before the trial and repeatedly sampled during the trial, first of which was done 24 h *post-inoculation* (*p*.*i*.). Groups using the breed Ross 308 (control group, increased amount of litter in the pen (I), and reduced stocking density (II)) were sampled for ten times during the 36 days of trial. The group using the breed Rowan x Ranger (III) was sampled 13 times during the 47 days of trial (sampling 1 = 24 h *p*.*i*., 2 = 72 h *p*.*i*., 3–5 = second week of trial, 6–7 = third week of trial, 8–9 = fourth week of trial, 10–11 = fifth week of trial, 12–13 = sixth week of trial (Fig 1)). To evaluate the colonization status of the broiler chickens, the swabs were immediately transferred into reaction tubes containing 500 μl PBS, thoroughly vortexed, and 50 μl were streaked out onto the chromogenic agar set as described above.

#### Colonization status at necropsy

A necropsy was performed at a target weight of two kilograms for all sampled seeder- and sentinel-birds (= d 36 (control, I-II) and d 47 (III); Fig 1). For sedation, ketamine hydrochloride (43 mg/kg body weight, Ketamin 10%, Bremer Pharma GmbH, Warburg, Germany), xylazine hydrochloride (1.75 mg/kg body weight, Xylavet 20mg/ml, cp-pharma, Burgdorf, Germany), and midazolam hydrochloride (0.85 mg/kg body weight, Midazolam 5mg/ml, Braun, Melsungen, Germany) were administered into the pectoral muscle. Animals were sacrificed by an intracardiac injection of tetracaine hydrochloride, mebezoniom iodide, and embutramid (1 ml/kg, T61, Intervet Deutschland GmbH, Germany). Digestive tract samples of crop, jejunum, cecum, and colon were quantitatively analyzed, and organ samples of liver and spleen were qualitatively analyzed for the occurrence of both bacterial strains as described in Robé et al. [12]. In brief, the digestive tract content was weighed in a reaction tube into which PBS was added at the ratio of 1:2. A dilution series in PBS was performed, and appropriate dilutions were plated on the plate set as described above. For quantification, a minimum of two dilution levels were counted. In addition, to exclude the possibility of systemic spread, organ samples of liver and spleen were investigated qualitatively as described before [12].

### Statistical analysis

Statistical analysis was carried out using SPSS Statistics 25 (IBM, New York, USA). Confidence intervals of proportions were calculated using the Clopper-Pearson method. For all analyses, the probability level to denote significance was set to 0.05.

The effects of the tested management measures on the colonization of broiler chickens with ESBL- and pAmpC- producing *E*. *coli* during the trials were analyzed via a logistic mixed regression model. The variables ‘group’, ‘sampling time’, and ‘animal’ were included in the analysis for both *E*. *coli* strains. To account for the repeated measurement of the same individual, ‘ID number’ and ‘sampling time’ were included in the analysis as random effects. Using backward selection, the best fitting model from a full model, including 2- and 3-way interactions, was obtained. The model with the lowest AIC value and lowest number of included effects was chosen, with an AIC change of two or less considered as equal. It resulted in the model with all main factors and the interaction term ‘animal and sampling time’. Residuals were inspected for normality and homoscedasticity for each sampling time point.

Mann-Whitney-U-Tests were performed to compare the level of colonization between the seeder- and sentinel-birds at necropsy. Kruskal-Wallis-Tests were performed to compare the final colonization of the digestive tracts of the tested groups to the control group at necropsy. Due to multiple comparisons in the Kruskal-Wallis-Test, the level of significance was set to 0.0167 (Bonferroni correction).

## Results

### Cloacal swabs

**Fig 2:**
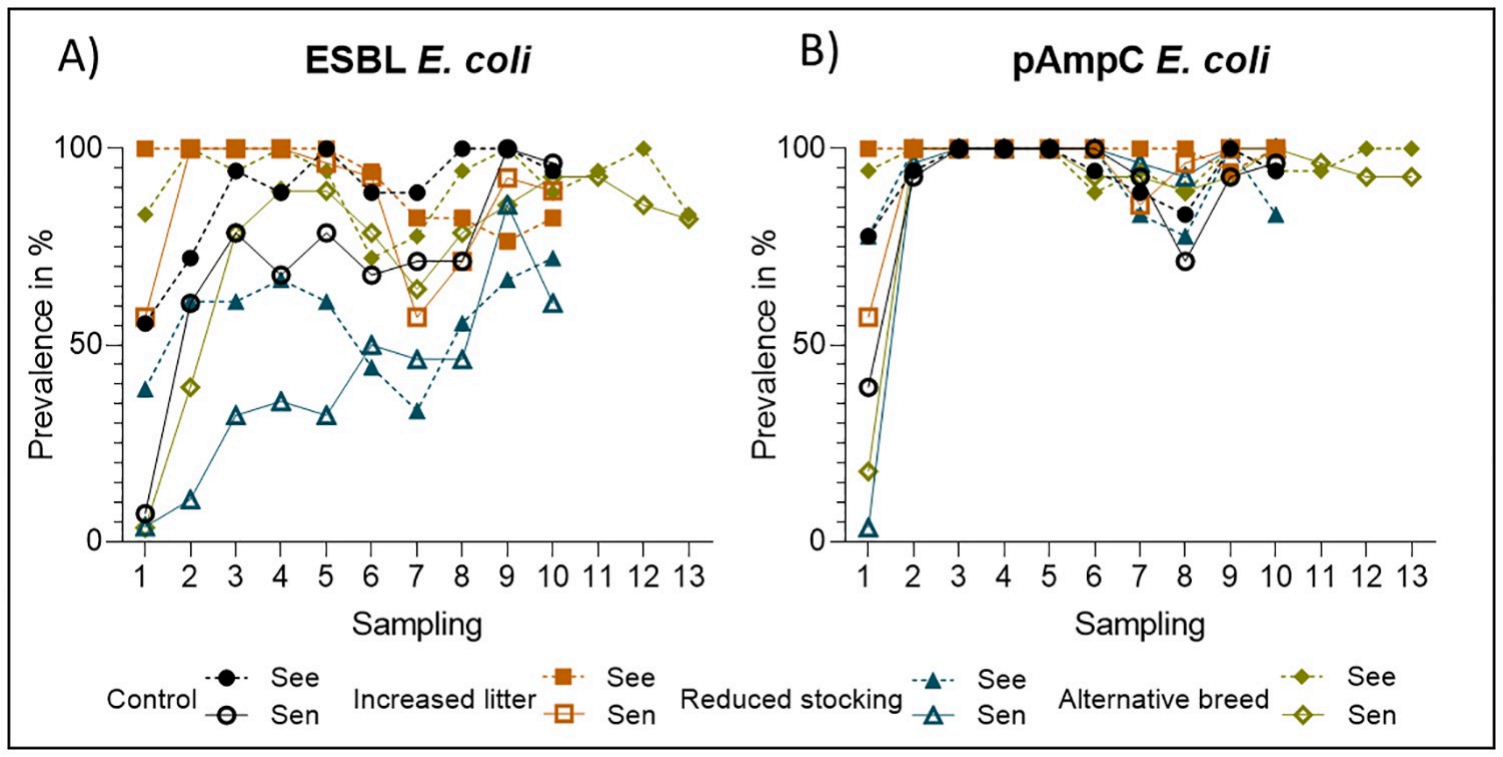
Prevalence of broiler chickens with (A) ESBL- and (B) pAmpC- producing *E*. *coli* throughout the different trials (control group, increased litter, reduced stocking density (reduced stocking) and alternative breed). See = seeder-birds, Sen = sentinel-birds; sampling 1 = 24 h post-inoculation, 2 = 72 h post-inoculation, 3–5 = 2^nd^ week of trial, 6–7 = 3^rd^ week of trial, 8–9 = 4^th^ week of trial, 10–11 = 5^th^ week of trial, 12–13 = 6^th^ week of trial.

Colonization of broiler chickens with the ESBL- and pAmpC- *E*. *coli* strains was monitored via cloacal swabs throughout each trial, starting 24 h *p*.*i*. A strain-dependent course of colonization was evident in the investigated groups (Fig 2, S1 Fig and S1 Table). ‘Group’, ‘animal’, ‘sampling time’ as well as the interaction between ‘animal and sampling time’ were statistically significant (Table 1). Compared to the control group for the ESBL- *E*. *coli* strain, a reduced hazard of colonization was solely apparent under the reduction of stocking density to 25 kg/m^2^, with a hazard ratio (HR) of 0.18 (95% CI: 0.12–0.28). An increased amount of litter in the pen led to a higher risk of colonization for both *E*. *coli* strains (ESBL- *E*. *coli* HR 2.38 (95% CI: 1.49–3.82); pAmpC- *E*. *coli* HR 4.58 (95% CI: 2.09–10.06)), while the use of an alternative breed showed no effect on the hazard of colonization of broiler chickens with both *E*. *coli* strains (ESBL- *E*. *coli* HR 1.01 (95% CI: 0.66–1.56); pAmpC- *E*. *coli* HR 1.25 (95% CI: 0.65–2.40)).

**Table 1:** Hazard ratios (HR) of broiler chickens’ colonization with ESBL- and pAmpC- producing *E*. *coli* in the four investigated groups (control group, increased litter, reduced stocking density (reduced stocking) and alternative breed) adjusted for interaction ‘animal and sampling time’.

Strain	Factor	*p*-value		HR (95% CI)
**ESBL- *E*. *coli***	Group	< 0.001	Control (reference)	1
			Increased litter (3 kg/m^2^)	2.38 (1.49–3.82)
			Reduced Stocking (25 kg/m^2^)	0.18 (0.12–0.28)
			Alternative breed (Rowan x Ranger)	1.01 (0.66–1.56)
	Animal	< 0.001	Seeder (reference)	1
			Sentinel	0.11 (0.05–0.23)
	Sampling time	< 0.001	1 (reference)	1
			2	3.90 (1.68–9.10)
			3	6.46 (2.57–16.25)
			4	7.54 (2.91–19.54)
			5	6.49 (2.57–16.36)
			6	2.36 (1.06–5.26)
			7	1.82 (0.83–3.97)
			8	4.35 (1.83–10.34)
			9	5.58 (2.26–13.75)
			10	4.91 (2.03–11.87)
**pAmpC- *E*. *coli***	Group	< 0.001	Control (reference)	1
			Increased litter (3 kg/m^2^)	4.58 (2.09–10.06)
			Reduced Stocking (25 kg/m^2^)	0.94 (0.50–1.78)
			Alternative breed (Rowan x Ranger)	1.25 (0.65–2.40)
	Animal	> 0.999	Seeder (reference)	not estimated
			Sentinel	
	Sampling time	< 0.001	1 (reference)	1
			2	10.56 (1.29–86.71)
			3	not estimated
			4	not estimated
			5	not estimated
			6	3.41 (0.86–13.48)
			7	1.61 (0.53–4.93)
			8	1.01 (0.36–2.80)
			9	10.83 (1.31–89.49)
			10	2.00 (0.62–6.49)

95% CI = 95% confidence interval; sampling time: 1 = 24 h *post inoculation*, 2 = 72 h *post inoculation*, 3–5 = 2nd week of trial, 6–7 = 3rd week of trial, 8–9 = 4th week of trial, 10 = 5th week of trial.

For the ESBL- *E*. *coli*, the hazard rate of colonization was lower for sentinel-birds compared to seeder-birds with a hazard ratio of 0.11 (95% CI: 0.05–0.23). A reduced hazard for sentinel-birds could not be shown for the pAmpC- *E*. *coli*, while the interaction between ‘animal and sampling time’ was highly significant (Table 1).

Except the seventh sampling time point, the hazard rates of colonization for the ESBL- *E*. *coli* strain were higher at all sampling times after 24 h. Regarding the pAmpC- *E*. *coli* strain, the hazard rates of colonization were higher at all sampling times after 24 h. However, statistical significance could only be shown for the second and ninth sampling time points due to strong interactions with the animal (Table 1).

### Necropsy

**Fig 3:**
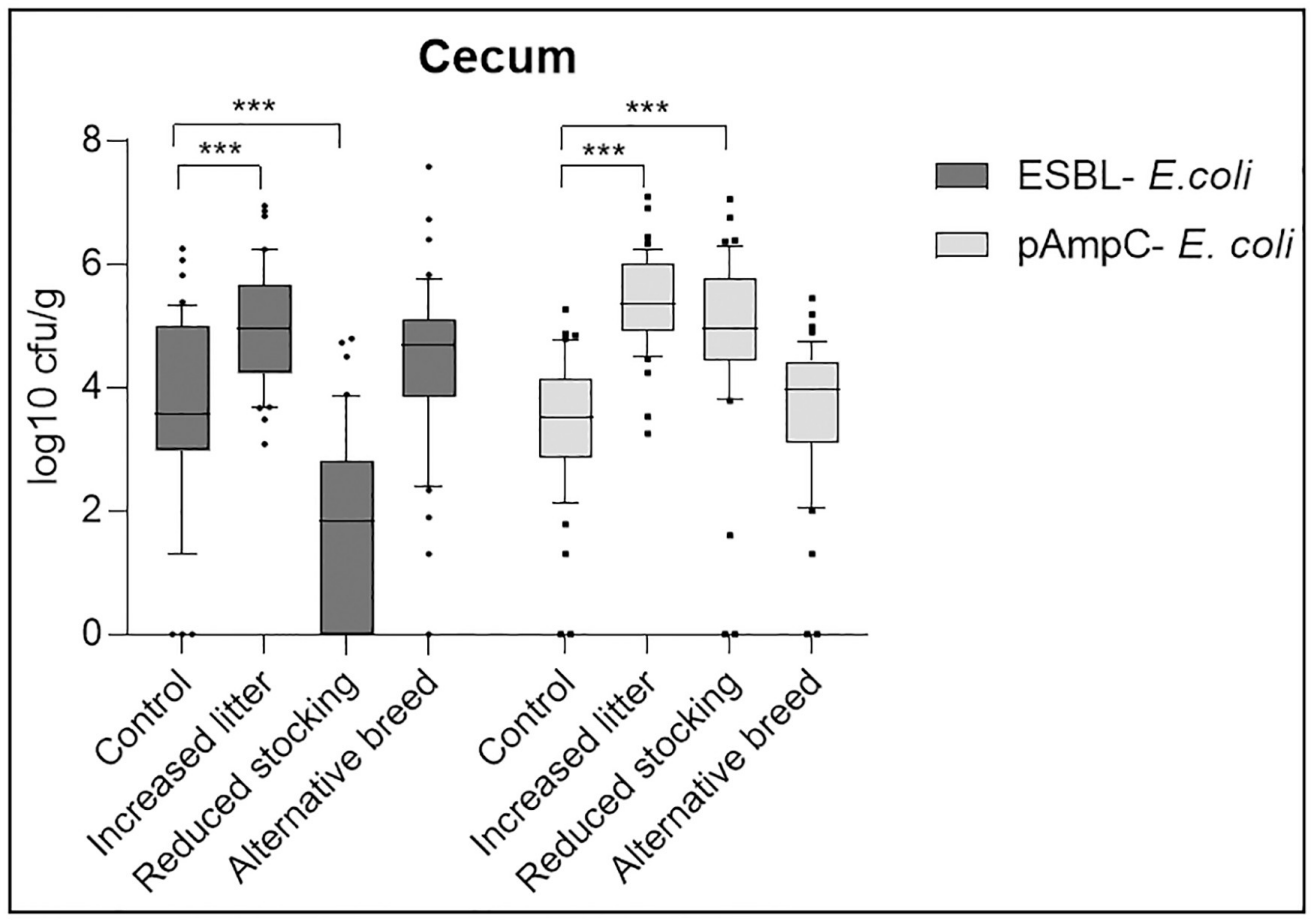
Cecum colonization of broiler chickens (log10 cfu/g) with ESBL- and pAmpC- producing *E*. *coli* in the four investigated groups (control group, increased litter, reduced stocking density (reduced stocking) and alternative breed) determined at necropsy. *** *p* < 0.001 (Kruskal-Wallis-Test).

At necropsy, respective contents of crop, jejunum, cecum, and colon were analyzed. As there was no significant difference in the level of colonization between the seeder- and sentinel-birds in any of the experimental groups (Mann-Whitney-U-Test, *p* > 0.05, S2 Table), each digestive tract sample per group was analyzed jointly for all investigated birds. None of the applied measures significantly reduced the bacterial counts of both the ESBL- and the pAmpC- *E*. *coli* strains in any of the digestive tract samples compared to the control group (Kruskal-Wallis-Test, Fig 3 and S2 Fig). We focused on the cecum samples for the following analyses as the results of all digestive tract samples correlated (Fig 3 and S2 Fig). By the reduction of the stocking density to 25 kg/m^2^, a strain-dependent significant reduction of cecum colonization of the ESBL- *E*. *coli* (Kruskal-Wallis-Test, *p* < 0.001, Fig 3) with a considerably lower prevalence of 63% (95% CI: 48–77%) compared to the control group (93%; 95% CI: 82–99%) was observed (S1 Table). On the other hand, the bacterial counts for the pAmpC- *E*. *coli* in the group with the reduced stocking density were significantly higher (Kruskal-Wallis-Test, *p* < 0.001, Fig 3) compared to the control group, with almost all broiler chickens pAmpC-colonized (96%; 95% CI: 85–99%, S1 Table). A significant increase in the cecum colonization with both bacterial strains (Kruskal-Wallis-Test, *p* < 0.001, Fig 3) was evident for the experimental group with the measure of a tripled amount of litter in the pen. The alternative broiler breed Rowan x Ranger caused no effect on the colonization with both bacterial *E*. *coli* stains (Kruskal-Wallis-Test, *p* > 0.05, Fig 3). These results are correlating to the analyses of the cloacal swabs (except the increased pAmpC- *E*. *coli* colonization in the group with reduced stocking density).

In summary, none of the tested measures prevented the colonization of broiler chickens with both the ESBL- and the pAmpC- producing *E*. *coli* strains. There was a significant reduction of the prevalence and the bacterial counts of the ESBL- *E*. *coli* in the group with the reduced stocking density. Besides, we did not detect colonization of the liver or spleen with our bacterial strains in any of our experimental groups.

## Discussion

In our experiments, none of the applied intervention measures reduced the broiler chickens’ colonization with both the ESBL- and the pAmpC- producing *E*. *coli* strains. All measures were tested separately under controlled conditions in a setup close to the real farming conditions, according to Robé et al. [12]. To the best of our knowledge, only little information about trials having our tested management measures to reduce the broiler chickens’ colonization with ESBL- and pAmpC- producing bacteria is available.

A study by Guardia et al. [24] demonstrated an effect of high stocking densities on the composition of commensal bacteria in the digestive tracts of young broilers. A decrease in the overall bacteria and *E*. *coli* in the chickens’ ceca at the age of three weeks followed by a reduced effect at the age of six weeks, was shown. Also, reduced stocking densities have been shown to affect the colonization of different bacteria, including pathogens such as *Clostridium perfringens*, *Campylobacter* spp. and *Salmonella* spp. [25–27]. A reasonable explanation is that the reduced bacterial contamination per square meter of the litter caused the effect on colonization [24]. As the intestinal microbiota of broiler chickens is affected by the composition, type, and quality of the litter [28, 29], an improved litter quality through less bacterial contamination could also lead to a reduced load of ESBL- and pAmpC- producing bacteria, particularly at the beginning of the fattening period. However, our results did not prove a general positive effect of reduced stocking density on the ESBL- and pAmpC- colonization of broiler chickens. On the one hand, we showed a significant reduction of cecum colonization of the ESBL- *E*. *coli*. On the other hand, a significant increase of the pAmpC- *E*. *coli* colonization of the broiler chickens was evident. Even though the co-colonization of different ESBL- and pAmpC- producing bacteria represents the real scenario of colonization [17], there is a lack of broiler studies using this approach. Based on our results, we hypothesize that different intervention measures have strain-dependent effects on the ESBL- and pAmpC- colonization of broiler chickens. A study by Nuotio et al. [15] described a variation in the effect of a commercial Competitive Exclusion culture on the broiler colonization with three separately tested ESBL- and pAmpC- *E*. *coli* strains. Thus, a combination of different approaches might be needed to reduce the spread of these resistant bacteria in the broiler production chain [17, 30].

A similar approach of less contact to contaminated feces was taken for the experimental group with increased amount of litter in the pen. We hypothesized that the fecal droppings which harbor the resistant bacterial strains mix in the bedding material, for example, due to the movement of the chickens. Conversely, the cecum colonization of both the ESBL- and the pAmpC- *E*. *coli* strains was significantly higher at necropsy compared to the control group. We assume that an increased amount of litter leads to a more pronounced explorative behavior of the broiler chickens with a higher oral intake of litter and results in an intensified (re-) colonization with the resistant bacteria [12]. The described quantities of bedding material used for broiler chickens range from 1 kg/m^2^ [31], which reflects the commercial standard in Germany and was applied in our control group, up to 6 kg/m^2^ [24] in some studies. However, because of practical reasons, a tripled amount of litter (3 kg/m^2^) was used in our related experimental group. A study by Persoons et al. [32] showed an impact of different litter materials on the occurrence of ceftiofur-resistant *E*. *coli* in broilers, with a higher risk for straw compared to wood curls. An effect of the bedding material on the ESBL- and pAmpC- colonization cannot be ruled out in our study, as all trials were conducted using pelletized straw granule. The fine litter structure of straw granule could cause a higher oral intake of the bedding material compared to other materials and a higher oral intake could have led to a higher (re-) colonization of the broiler chickens in our trial. As we want to mimic real farming conditions, we used pelletized straw granule which is most frequently used as bedding material in broiler fattening farms in Germany. As fresh litter is known to carry enteral bacteria [33], the absence of ESBL- and pAmpC- producing bacteria was confirmed before the trials. In order to reflect real farming conditions to the best of our ability, we did not sterilize the litter. Hence, an interaction between the broiler chickens and the surrounding environment including the present bacterial spectrum was possible as usual in broiler production [34].

An impact of the broiler breed on the microbial composition and colonization of the gut with resistant or pathogen bacteria is discussed [32, 35–37]. A strong influence of the environment on the microbial composition with a minor impact of the used broiler breed was concluded by Richards et al. [38]. Contrastingly, Schokker et al. [39] drew a different conclusion with a major influence of broilers’ genetic on microbial gut colonization. These diverse results of the two exemplary mentioned studies could also be caused by the different experimental designs. While Richards et al. [38] placed all breeds together in one pen, Schocker et al. [39] housed the investigated breeds simultaneously, but separately, under identical conditions for the chickens. Furthermore, Rychlik [40] demonstrated a highly variable microbiota development even in the ceca of chickens of the same line, which had the same background but were not kept simultaneously. A general statement on the impact of the breed on broiler chickens’ gut colonization is not possible yet, due to the different housing conditions and a large variety of breeds used in various studies. In our trial, we placed the broiler breeds separately but under identical conditions. Investigating the broiler breeds Ross 308 and Rowan x Ranger, no significant difference in colonization with the ESBL- and the pAmpC- *E*. *coli* was apparent. Thus, our results support the findings of Richards et al. [38] even though we placed the two broiler breeds in separate pens.

Various scenarios can lead to ESBL- and pAmpC- colonization of broiler chickens, as reviewed by Dame-Korevaar et al. [11]. Transmission of antibiotic-resistant bacteria can take place before the arrival of the broiler chickens on the fattening farms [6] as well as after the placement of the chickens [7]. We investigated the impact of singularly applied management measures on the colonization of both the ESBL- and the pAmpC- inoculated seeder-birds and non-inoculated sentinel-birds. In our recently published broiler colonization model, a ratio of 1:5 ESBL- and pAmpC- inoculated seeder-birds to non-inoculated sentinel-birds on the third day of the trial led to the colonization of all seeder-birds 24 h *p*.*i*. and colonization of all sentinel-birds 72 h *p*.*i*. [12]. In contrast to our broiler colonization model, the resulting prevalence of the seeder- and sentinel-birds of our control group in the current study was lower, 24 h and 72 h *p*.*i*. The lower prevalence may be due to the differences in the group size, with 20 broiler chickens in the colonization model compared to 90 broiler chickens in the current study. A bigger group size results in a larger pen, which might have affected the group dynamics. Compared to the control group, the prevalence in the intervention groups might be due to an impact of the applied intervention measure itself or can partly be a result of the variability of cloacal swab samples. As fermentation of the bacteria takes place in the ceca [41], and the cecal content is ejected only twice a day [40], the detection rate of our colonization strains might be affected by the chosen sampling type. Stanley et al. [42] pointed out the need of cloacal swab samples from the same birds for a repeated measurement in a trial but also showed quantitative differences between the analysis results of cloacal swab samples and cecal samples. However, statistical analyses of the swab samples revealed results comparable to the results for cecal samples. Additionally, there was a correlation among the analysis results of crop-, jejunum-, colon-, and the cecum samples per experimental group. As the contents of crop and jejunum simply reflect the recent uptake of the resistant bacteria from the surrounding, they are strongly influenced by the eating and drinking behavior of the broilers immediately before the necropsy [12]. Nevertheless, a reasonable explanation for the correlating results for the different digestive tract samples is that the uptake of fecal droppings caused an oral (re-)colonization of the broiler chickens. Consequently, cecum samples are needed in order to have the ESBL- and pAmpC- status of the broilers evaluated [40].

To summarize, the effects of the tested management measures, namely (I) an increased amount of litter in the pen, (II) the reduction of stocking density to 25 kg/m^2^, and (III) the use of an alternative broiler breed, are negligible on the ESBL- and pAmpC- colonization of broiler chickens. Nevertheless, these results do not entirely exclude the possibility of management measures reducing the colonization of broiler chickens with ESBL- and pAmpC- producing bacteria. Particularly, microbiome-directed measures could represent a promising effect as they directly address the digestive tract. Probiotics such as commercial Competitive Exclusion cultures and phytobiotics already showed positive results of reducing the ESBL- and pAmpC- colonization of broiler chickens [15–18] and should be further investigated. In addition, a combination of promising measures is another approach which needs to be evaluated for a potential synergistic effect against the ESBL- and pAmpC- colonization of broiler chickens. Apart from the colonization of the broiler chickens, the external contamination with ESBL- and pAmpC- producing bacteria has to be considered as a source of contamination at slaughterhouse level. In terms of the consumer protection, investigations on the external contamination of broiler chickens are necessary.

## Supporting information

S1 Fig Prevalence of seeder-birds and sentinel-birds throughout all trials (control group, increased litter, reduced stocking density, and alternative breed) with (A) ESBL- and (B) pAmpC- producing *E*. *coli*. Samplings: 1 = 24 h post-inoculation, 2 = 72 h post-inoculation, 3–5 = 2^nd^ week of trial, 6–7 = 3^rd^ week of trial, 8–9 = 4^th^ week of trial, 10 = 5^th^ week of trial; Error bar = 95% Confidence interval.
(TIF)

S2 Fig Colonization of broiler chickens (log10 cfu/g) with ESBL- and pAmpC- producing *E*. *coli* in (A) crop, (B) jejunum, and (C) colon in the four investigated groups (control group, increased litter, reduced stocking density (reduced stocking) and alternative breed) determined at necropsy. *** *p* < 0.001 (Kruskal-Wallis-Test).
(TIF)

S1 Table Prevalence of ESBL- and pAmpC- producing *E*. *coli* of seeder-birds and sentinel-birds determined during the trial (cloacal swabs) and at necropsy of the four investigated groups (control group, increased litter, reduced stocking density (reduced stocking) and alternative breed). * Prevalence in percent (%); 10716 = ESBL- *E*. *coli*, 10717 = pAmpC- *E*. *coli*; See = seeder-birds, Sen = sentinel-birds; sampling: 1 = 24 h post-inoculation, 2 = 72 h post-inoculation, 3–5 = 2^nd^ week of trial, 6–7 = 3^rd^ week of trial, 8–9 = 4^th^ week of trial, 10–11 = 5^th^ week of trial, 12–13 = 6^th^ week of trial.
(DOCX)

S2 Table Mean values and confidence intervals of ESBL- and pAmpC- producing *E*. *coli* of digestive tract samples (crop, jejunum, cecum, and colon) of the four investigated groups (control group, increased litter, reduced stocking density (reduced stocking) and alternative breed) determined at necropsy. 10716 = ESBL- *E*. *coli*, 10717 = pAmpC- *E*. *coli*; all data shown are log10 transformed (log10 cfu/g); ± CI = ± 95% Confidence interval.
(DOCX)
