# Supplementary figures and images for: Impact of different management measures on the colonization of broiler chickens with ESBL- and pAmpC- producing Escherichia coli in an experimental seeder-bird model
Source: PLoS One. 2021 Jan 7;16(1):e0245224. doi: 10.1371/journal.pone.0245224 (PMC7790425; doi:10.1371/journal.pone.0245224)

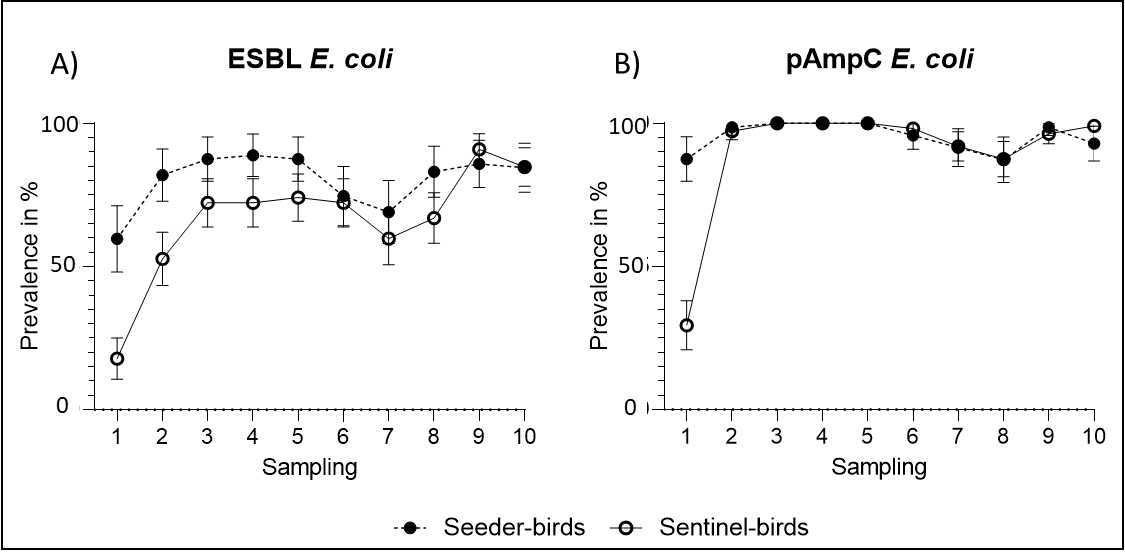

Supplement: S1 Fig — Samplings: 1 = 24 h post-inoculation, 2 = 72 h post-inoculation, 3–5 = 2nd week of trial, 6–7 = 3rd week of trial, 8–9 = 4th week of trial, 10 = 5th week of trial; Error bar = 95% Confidence interval. (TIF) [file pone.0245224.s001.tif]

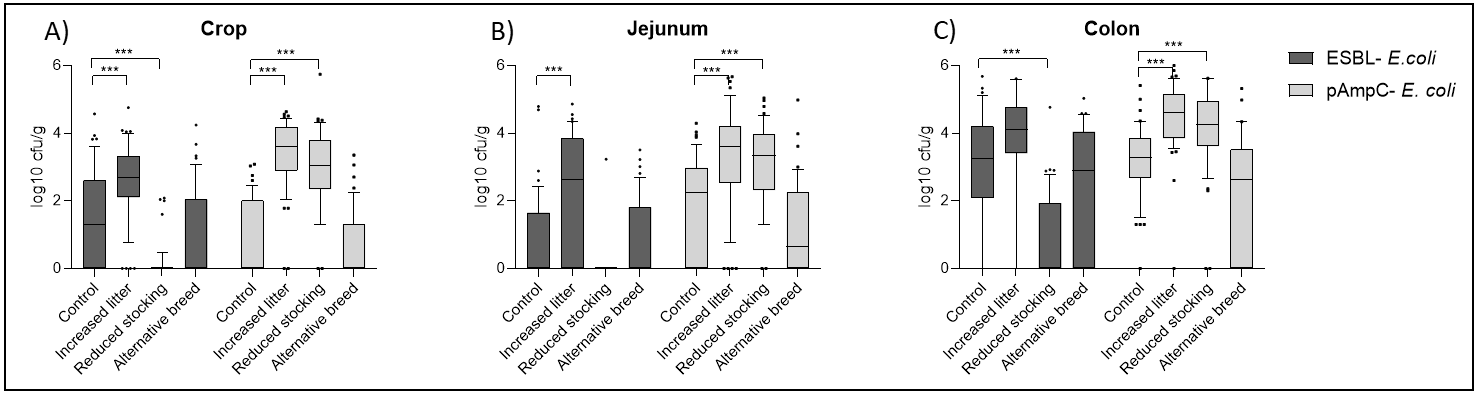

Supplement: S2 Fig — *** p < 0.001 (Kruskal-Wallis-Test). (TIF) [file pone.0245224.s002.tif]
