# Supplementary material for: Impact of different management measures on the colonization of broiler chickens with ESBL- and pAmpC- producing Escherichia coli in an experimental seeder-bird model
Source: PLoS One. 2021 Jan 7;16(1):e0245224. doi: 10.1371/journal.pone.0245224 (PMC7790425; doi:10.1371/journal.pone.0245224)
Supplement: S1 Table — * Prevalence in percent (%); 10716 = ESBL- E. coli, 10717 = pAmpC- E. coli; See = seeder-birds, Sen = sentinel-birds; sampling: 1 = 24 h post-inoculation, 2 = 72 h post-inoculation, 3–5 = 2nd week of trial, 6–7 = 3rd week of trial, 8–9 = 4th week of trial, 10–11 = 5th week of trial, 12–13 = 6th week of trial. (DOCX) [file pone.0245224.s003.docx]

**S1 Table. Prevalence of ESBL- and pAmpC- producing *E. coli* of seeder-birds and sentinel-birds determined during the trial (cloacal swabs) and at necropsy of the four investigated groups (Control group, Increased litter, Reduced stocking density (Reduced Stocking) and Alternative breed).**

| **Group** | **Control group** | | | | |  | **Increased litter**  **(3 kg/m^2^)** | | | |  | **Reduced stocking**  **(25 kg/m^2^)** | | | | |  | **Alternative breed**  **(Rowan x Ranger)** | | | | | | |
| --- | --- | --- | --- | --- | --- | --- | --- | --- | --- | --- | --- | --- | --- | --- | --- | --- | --- | --- | --- | --- | --- | --- | --- | --- |
| Strain | 10716 | | 10717 | | |  | 10716 | | 10717 | |  | 10716 | | | 10717 | |  | 10716 | | | 10717 | | | |
|  |  | |  | | |  |  | |  | |  |  | | |  | |  |  | | |  | | | |
| Animal | See* | Sen* | See* | Sen* | |  | See* | Sen* | See* | Sen* |  | See* | | Sen* | See* | Sen* |  | See* | | Sen* | See* | | Sen* | |
|  | | | | |  | | | | | | | |  | | | | | | | | |  | | |
| 1 | 56 | 7 | 78 | 39 | |  | 100 | 57 | 100 | 57 |  | 39 | | 4 | 78 | 4 |  | 83 | | 4 | 94 | | 18 | |
| 2 | 72 | 61 | 94 | 93 | |  | 100 | 100 | 100 | 100 |  | 61 | | 11 | 100 | 96 |  | 100 | | 39 | 100 | | 100 | |
| 3 | 94 | 79 | 100 | 100 | |  | 100 | 100 | 100 | 100 |  | 61 | | 32 | 100 | 100 |  | 94 | | 79 | 100 | | 100 | |
| 4 | 89 | 68 | 100 | 100 | |  | 100 | 100 | 100 | 100 |  | 67 | | 36 | 100 | 100 |  | 100 | | 89 | 100 | | 100 | |
| 5 | 100 | 79 | 100 | 100 | |  | 100 | 96 | 100 | 100 |  | 61 | | 32 | 100 | 100 |  | 94 | | 89 | 100 | | 100 | |
| 6 | 89 | 68 | 94 | 100 | |  | 94 | 93 | 100 | 100 |  | 44 | | 50 | 100 | 100 |  | 72 | | 79 | 89 | | 93 | |
| 7 | 89 | 71 | 89 | 93 | |  | 82 | 57 | 100 | 86 |  | 33 | | 46 | 83 | 96 |  | 78 | | 64 | 94 | | 93 | |
| 8 | 100 | 71 | 83 | 71 | |  | 82 | 71 | 100 | 96 |  | 56 | | 46 | 78 | 93 |  | 94 | | 79 | 89 | | 89 | |
| 9 | 100 | 100 | 100 | 93 | |  | 77 | 93 | 94 | 100 |  | 67 | | 86 | 100 | 100 |  | 100 | | 86 | 100 | | 93 | |
| 10 | 94 | 96 | 94 | 96 | |  | 82 | 89 | 100 | 100 |  | 72 | | 39 | 83 | 100 |  | 89 | | 93 | 94 | | 100 | |
| 11 | - | - | - | - | |  | - | - | - | - |  | - | | - | - | - |  | 94 | | 93 | 94 | | 96 | |
| 12 | - | - | - | - | |  | - | - | - | - |  | - | | - | - | - |  | 100 | | 86 | 100 | | 93 | |
| 13 | - | - | - | - | |  | - | - | - | - |  | - | | - | - | - |  | 83 | | 82 | 100 | | 93 | |
| *Necropsy* |  |  |  |  | |  |  |  |  |  |  |  | |  |  |  |  |  | |  |  | |  | |
| Crop | 56 | 50 | 39 | 50 | |  | 76 | 93 | 88 | 100 |  | 17 | | 4 | 94 | 96 |  | 39 | | 50 | 28 | | 36 | |
| (See+Sen) | (52) | | (46) | | |  | (87) | | (96) | |  | (9) | | | (96) | |  | (46) | | | (33) | | | |
|  |  | |  | | |  |  | |  | |  |  | | |  | |  |  | | |  | | | |
| Jejunum | 33 | 29 | 72 | 75 | |  | 65 | 75 | 94 | 89 |  | 6 | | 0 | 94 | 96 |  | 33 | 46 | | 50 | | | 50 |
| (See+Sen) | (30) | | (74) | | |  | (71) | | (91) | |  | (2) | | | (96) | |  | (41) | | | (50) | | | |
|  |  | |  | | |  |  | |  | |  |  | | |  | |  |  | | |  | | | |
| Cecum | 94 | 93 | 94 | 96 | |  | 100 | 100 | 100 | 100 |  | 67 | | 61 | 94 | 96 |  | 100 | 96 | | 94 | | | 96 |
| (See+Sen) | (93) | | (96) | | |  | (100) | | (100) | |  | (63) | | | (96) | |  | (98) | | | (96) | | | |
|  |  | |  | | |  |  | |  | |  |  | | |  | |  |  | | |  | | | |
| Colon | 78 | 89 | 100 | 96 | |  | 94 | 82 | 100 | 96 |  | 56 | | 25 | 89 | 100 |  | 39 | 79 | | 50 | | | 75 |
| (See+Sen) | (85) | | (98) | | |  | (87) | | (98) | |  | (37) | | | (96) | |  | (63) | | | (65) | | | |
|  |  | |  | | |  |  | |  | |  |  | | |  | |  |  | | |  | | | |

* Prevalence in percent (%); 10716 = ESBL- *E. coli*, 10717 = pAmpC- *E. coli*; See = seeder-birds, Sen = sentinel-birds; sampling: 1 = 24 h post-inoculation, 2 = 72 h post-inoculation, 3-5 = 2^nd^ week of trial, 6-7 = 3^rd^ week of trial, 8-9 = 4^th^ week of trial, 10-11 = 5^th^ week of trial, 12-13 = 6^th^ week of trial
