# Supplementary material for: Impact of different management measures on the colonization of broiler chickens with ESBL- and pAmpC- producing Escherichia coli in an experimental seeder-bird model
Source: PLoS One. 2021 Jan 7;16(1):e0245224. doi: 10.1371/journal.pone.0245224 (PMC7790425; doi:10.1371/journal.pone.0245224)
Supplement: S2 Table — 10716 = ESBL- E. coli, 10717 = pAmpC- E. coli; all data shown are log10 transformed (log10 cfu/g); ± CI = ± 95% Confidence interval. (DOCX) [file pone.0245224.s004.docx]

**S2 Table. Mean values and confidence intervals of ESBL- and pAmpC- producing *E. coli* of digestive tract samples (crop, jejunum, cecum, and colon) of the four investigated groups (Control group, Increased litter, Reduced stocking density (Reduced stocking) and Alternative breed) determined at necropsy.**

| **Sample** |  | **Crop** | | | |  | **Jejunum** | | | |  | **Cecum** | | | |  | **Colon** | | | |
| --- | --- | --- | --- | --- | --- | --- | --- | --- | --- | --- | --- | --- | --- | --- | --- | --- | --- | --- | --- | --- |
| Strain |  | 10716 | | 10717 | |  | 10716 | | 10717 | |  | 10716 | | 10717 | |  | 10716 | | 10717 | |
|  |  | Mean | ± CI | Mean | ± CI |  | Mean | ± CI | Mean | ± CI |  | Mean | ± CI | Mean | ± CI |  | Mean | ± CI | Mean | ± CI |
| **Control group**  Seeder-birds  Sentinel-birds | | 1.18  1.50 | 0.66  1.71  0.89  2.10 | 0.78  1.03 | 0.03  1.04  0.62  1.43 |  | 0.80  0.67 | 0.20  1.39  0.23  1.12 | 1.88  2.00 | 1.28  2.48  1.49  2.51 |  | 3.70  3.57 | 3.00  4.32  2.98  4.16 | 3.51  3.35 | 2.95  4.07  2.97  3.74 |  | 2.84  3.07 | 2.00  3.68  2.50  3.63 | 3.41  3.03 | 2.89  3.93  2.68  3.37 |
| **Increased litter**  **(3 kg/m^2^)**  Seeder-birds  Sentinel-birds | | 2.42  2.62 | 1.87  2.97  2.21  3.03 | 3.16  3.51 | 2.56  3.77  3.20  3.82 |  | 2.44  2.25 | 1.54  3.35  1.67  2.83 | 3.49  3.24 | 2.83  4.15  2.71  3.77 |  | 4.70  5.12 | 4.39  5.09  4.76  5.48 | 5.24  5.51 | 4.89  5.59  5.21  5.80 |  | 4.06  3.54 | 3.43  4.69  2.86  4.21 | 4.51  4.43 | 4.23  4.79  3.99  4.86 |
| **Reduced stocking**  **(25 kg/m^2^)**  Seeder-birds  Sentinel-birds | | 0.31  0.07 | 0.00  0.64  0.00  0.22 | 2.95  2.95 | 2.42  3.47  2.54  3.36 |  | 0.18  0.00 | 0.00  0.52  0.00  0.00 | 2.91  3.19 | 2.30  3.53  2.80  3.58 |  | 1.75  1.68 | 1.10  2.39  1.09  2.27 | 4.84  4.85 | 4.06  5.62  4.41  5.29 |  | 1.09  0.70 | 0.59  1.58  0.22  1.17 | 4.03  4.19 | 3.23  4.82  3.83  4.54 |
| **Alternative breed**  **(Rowan x Ranger)**  Seeder-birds  Sentinel-birds | | 1.18  1.01 | 0.47  1.89  0.60  1.43 | 0.63  0.62 | 0.31  1.13  0.29  0.96 |  | 1.27  0.97 | 0.60  1.94  0.55  1.38 | 0.74  1.11 | 0.22  1.26  0.64  1.57 |  | 3.96  4.75 | 3.37  4.55  4.26  5.23 | 3.68  3.64 | 3.04  4.32  3.28  4.00 |  | 1.34  2.93 | 0.50  2.28  2.17  3.58 | 1.72  2.48 | 0.78  2.65  1.88  3.09 |

10716 = ESBL- *E. coli*, 10717 = pAmpC- *E. coli*; all data shown are log10 transformed (log10 cfu/g); ± CI = ± 95% Confidence interval
